# Cytotoxicity and Antimicrobial Activity of Oral Rinses In Vitro

**DOI:** 10.1155/2017/4019723

**Published:** 2017-03-19

**Authors:** Heinz-Dieter Müller, Sigrun Eick, Andreas Moritz, Adrian Lussi, Reinhard Gruber

**Affiliations:** ^1^Department of Preventive, Restorative and Pediatric Dentistry, School of Dental Medicine, University of Bern, Bern, Switzerland; ^2^Department of Oral Biology, Medical University of Vienna, Vienna, Austria; ^3^Department of Periodontology, School of Dental Medicine, University of Bern, Bern, Switzerland; ^4^Department of Conservative Dentistry and Periodontology, Medical University of Vienna, Vienna, Austria

## Abstract

While oral rinses used for cosmetic purposes only do not necessarily have to be antiseptic, antimicrobial activity is required for medical indications, including oral and periodontal surgery. So the question arises—is the antimicrobial activity of oral rinses associated with any destructive changes in cell viability in vitro? To answer this question, we examined twelve oral rinses with respect to their antimicrobial and cytotoxic activity. Antimicrobial activity was screened against five bacterial strains using disc diffusion. Cytotoxicity was determined by mitochondrial reductase activity with primary gingival fibroblasts, L929 cells, and HSC-2 epithelial cells. Phase contrast microscopy and trypan blue staining were then performed to reveal cell morphology. Cells remained vital after exposure to oral rinses that were only used for cosmetic purposes. Moderate cytotoxic effects were observed for oral rinses containing 0.05% chlorhexidine, ethanol, or pegylated hydrogenated castor oil and sodium dodecyl sulfate. Other oral rinses containing 0.2% chlorhexidine and cocamidopropyl betaine exhibited strong cytotoxic and antimicrobial activity. Strong cytotoxic but moderate antimicrobial activity was observed in oral rinses containing cetylpyridinium chloride. The in vitro data show that oral rinses are heterogeneous with respect to their cytotoxic and antimicrobial effects. Based on their respective properties, oral rinses can be selected either to reduce the microbial load or for cosmetic purposes.

## 1. Introduction

Oral rinses, also called mouthwashes, are most often used to reduce the microbial load in the oral cavity or to control or reduce bad breath, depending on the ingredients in the particular oral rinse [1]. The antimicrobial activity is used to control supragingival plaque and gingivitis, and it is utilized prior to oral and periodontal surgery, including tooth extraction and implant placement [2]. Cosmetic oral rinses are not necessarily antimicrobial, but they do provide an agreeable taste and reduce halitosis [3]. The oral mucosa comes in contact with the oral rinse when the oral cavity is flushed [4]. Particularly in cases of injury, the oral rinse also comes in contact with the underlying gingival connective tissue [4]. Even though side effects from the use of oral rinses are rare, allergic reactions to defined ingredients are occasionally reported [5, 6]. It is likely that these rare events have led to preclinical and, in particular, in vitro testing of oral rinses with the aim of understanding if oral rinses have an antimicrobial activity and whether they are ultimately cytotoxic, at least under in vitro conditions.

Representative standard formulations of oral rinses are supplemented with chlorhexidine digluconate, ethanol, essential oils, and detergents [7, 8]. Oral rinses can contain anti-inflammatory glucocorticoids and aloe vera, anesthetics such as lidocaine and morphine, antifungal nystatin, antihistamine diphenhydramine, and antimicrobials [9–11]. Oral rinses also contain chemicals, glycerin as a humectant, sodium benzoate as a buffer, flavors, coloring, and emulsifiers that serve as stabilizers. In summary, oral rinses are loaded with various substances that are described as supporting oral health and therefore preventing or decreasing the severity of oral diseases [12, 13]. However, despite the evidence that single ingredients of oral rinses are cytotoxic and effective against bacteria, the impact of the complex cocktail of supplements on oral cell cytotoxicity and antimicrobial activity is largely unknown.

Evidence has been accumulated that chlorhexidine (CHX) gluconate-containing oral rinses have antibacterial activity and reduce bacteria on buccal epithelia and pellicles [14]. Several microorganisms, such as* Actinomyces naeslundii*,* Veillonella dispar*, and* Prevotella nigrescens*, and the streptococci are highly susceptible to CHX [15]. CHX is also cytotoxic, as reported for human gingival fibroblasts and osteosarcoma cells [16]. Ethanol is partially effective against oral microorganisms and showed antimicrobial activity against planktonic* Streptococcus mutans* cells on the surface of orthodontic brackets [17]. Sodium dodecyl sulfate, however, is a detergent with no reported antibacterial activity [18]. Other components, such as pegylated hydrogenated castor oils, are detergents used in the cosmetic industry with no reported antibacterial activity [19] but a negative impact on keratinocyte viability [20]. Cetylpyridinium chloride possesses antimicrobial activity, is used in oral rinses [21, 22], and has a negative impact on L929 fibroblast viability [23]. Thus, oral rinses can have antimicrobial activity, and since detergents usually disrupt cell membranes, toxicity for oral cells is of potential concern. Until now, oral rinse studies have not provided information about antibacterial effects and viability changes of oral mucosa cells.

The aim of this study was to investigate the antibacterial effects and cytotoxicity of oral rinses. Therefore, three different cell lines, including primary gingival fibroblasts, epithelial cells, and L929 cells, were exposed to a dozen different oral rinses. Antibacterial effects were investigated using five different oral microorganisms associated with periodontal disease.

## 2. Materials and Methods

### 2.1. Cell Cultures

Human oral fibroblasts were obtained from healthy patients who had no record of periodontal disease. Patients gave their informed and written consent. An ethical statement of approval was obtained from the ethical committee of the Medical University of Vienna (EK NR 631/2007) as well. Oral fibroblasts were cultured in Dulbecco's modified Eagle medium (DMEM, Invitrogen Corporation, Carlsbad, CA, USA) supplemented with 10% fetal bovine serum (Life Technologies, Carlsbad, CA, USA) and antibiotics (Life Technologies) at 37°C, 5% CO_2_, and 95% humidity. For indicated experiments, human oral epithelial carcinoma cells (HSC-2) and murine aneuploid fibrosarcoma cells (L929) were used. In total, three strains of oral fibroblasts were established, and cultures of less than 10 passages were used for the experiments. For all experiments, cells were seeded at a concentration of 30,000 cells/cm^2^ onto culture dishes one day prior to stimulation.

### 2.2. Screening of Antimicrobial Activity

For the antimicrobial activity experiments, the following strains were used:* Actinomyces naeslundii* ATCC 12104,* Streptococcus gordonii* ATCC 10558,* Porphyromonas gingivalis* ATCC 33277,* Fusobacterium nucleatum* ATCC 25586, and* Tannerella forsythia* ATCC 43037. Additionally, a mixed population that included all five strains was investigated. The strains were* Streptococcus gordonii* cultured for 24 to 72 hours prior to the experiments on tryptic soy agar. All strains were grown anaerobically at 37°C except for* A. naeslundii* and* Strep. gordonii,* which were cultured in 5% CO_2_ aerobic conditions. A microbial suspension was prepared from all cultures of McFarland standard 0.5 (~1.5 × 10^8^ microorganisms) in 0.9% w/v sodium chloride solution and diluted 1 to 10. For the mixed population, 25 *μ*l of* Strep. gordonii* suspension, 50 *μ*l of* A. naeslundii* suspension, and 100 *μ*l each of the suspensions of the other strains were mixed together before being diluted 1 to 10. The different *μ*l volumes were used to avoid streptococci inhibiting anaerobic bacterial growth, establishing a biofilm containing all investigated bacteria [24].

For analyzing the antimicrobial activity of tested oral rinses, a modified agar diffusion method was used as follows: 100 *μ*l of the bacterial suspensions described above was spread on agar plates supplemented with 5% blood (Wilkins Chalgren agar, Oxoid, Basingstoke, GB). Thereafter, test filter disks without coating (BD Franklin Lakes, NJ, USA) were added to the agar plates, and 10 *μ*l of each oral rinse was pipetted on a separate filter disk. For controls, ethanol (10–20%), chlorhexidine (0.05–0.2%; CHX), cetylpyridinium chloride (0.01–0.05%; CPC), pegylated hydrogenated castor oils (0.1–10%; Cremophor® RH 40; PEG), sodium dodecyl sulfate (0.25–1.0%; SDS), and SF (125–500 ppm) were used (all Sigma-Aldrich, St. Louis, MO). After incubation took place at 37°C in the indicated atmosphere for 18 to 42 hours, the inhibition zones were measured [24].

### 2.3. Stimulation of Gingival Fibroblasts

Oral fibroblasts were exposed to a series of dilutions of 12 different oral rinses for 2 minutes and 24 hours. The oral rinses were randomly selected and investigated. The 12 oral rinses are as follows: #1 Mundspülung Hauschka (WALA Heilmittel, Germany), #2 Emofluor (Dr. Wild, Switzerland), #3 Parodentosan oral rinse (Tentan, Switzerland), #4 Listerine Total Care (Johnson & Johnson, UK), #5 Tebodont Mundspülung (Dr. Wild, Switzerland), #6 Dontodent (dm-drogerie markt, Austria), #7 Meridol and #8 Meridol med (GABA, Switzerland), #9 Sensodyne (GlaxoSmithKline, UK), #10 One Drop Only, Germany, #11 Elmex Sensitive Professional, and #12 Elmex professional erosion (GABA, Switzerland) (Table 1). Thereafter, viability assays were performed. Viability was determined via a formazan formation assay (Sigma, St. Louis, USA) and upon visual inspection via phase contrast microscopy and trypan blue staining [25].

### 2.4. Statistical Analysis

For calculation of lethal concentration (LC50), linear interpolation was performed in five independent experiments. For the antimicrobial activity, three independent replicates were analyzed, all via GraphPad Prism 6.0 (GraphPad Software Inc., San Diego, USA).

## 3. Results

### 3.1. Oral Rinses Have a Heterogeneous Impact on Oral Cell Viability In Vitro

**Figure 1:**
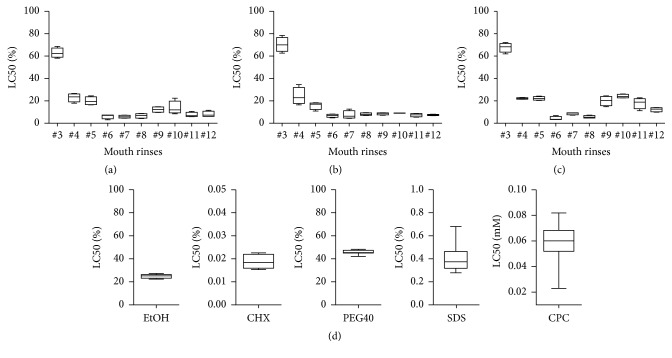
Lethal concentration (LC) 50 determined using a series of oral rinse dilutions and oral rinse additives applied to gingival fibroblasts and L929 and HSC-2 cells. (a, d) Gingival fibroblasts, (b) L929 cells, and (c) HSC-2 cells were cultured overnight and exposed to selected oral rinse dilutions and oral rinse additives for 2 minutes. Cell viability was measured with a formazan formation assay. Statistical analysis was performed, and LC50 was calculated. Graphs show a box plot, for example, the box represents the first and third quartiles, the median separating the higher half of a data from the lower half, and the whiskers indicate the minimum and maximum of the data. For oral rinses #1 and #2, cells remained vital; therefore the LC50 was not calculable and was excluded. For all experiments, cells with less than 10 passages were used. At least five independent experiments for each cell line were performed.

**Figure 2:**
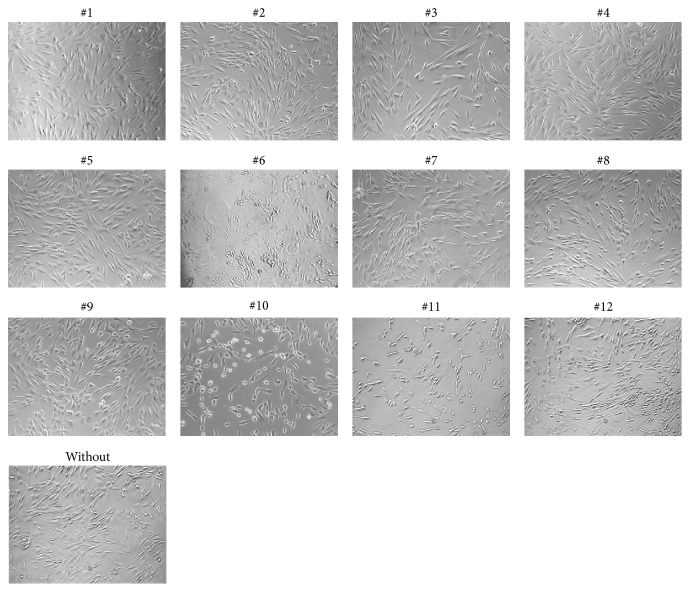
Phase contrast microscopy of gingival fibroblasts exposed to different oral rinses. Cultured gingival fibroblasts were exposed to a tenfold dilution of a panel of oral rinses for 2 minutes. Afterwards, cell morphology was analyzed via phase contrast microscopy. A physiological bipolar cell shape was observed in #1 to #5, which turned into a round cell shape in #7 to #12. Cell detachment was observed after exposure to oral rinse #6. Images were taken at a tenfold magnification.

**Figure 3:**
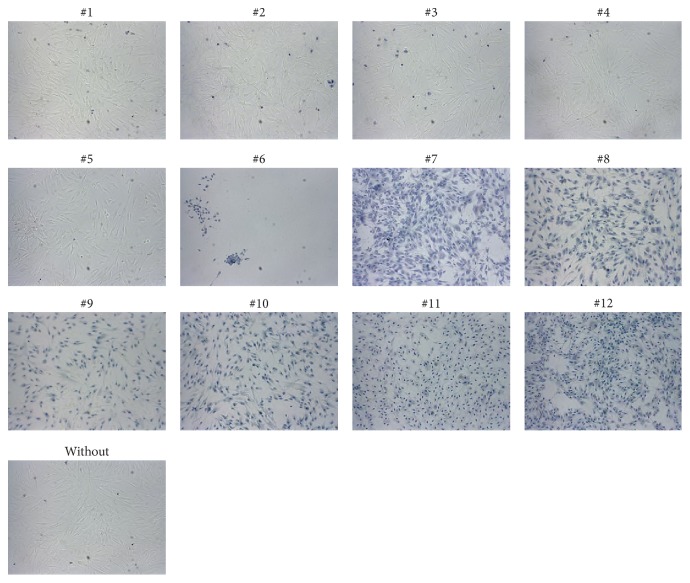
Microscopy of trypan blue-stained gingival fibroblasts exposed to different oral rinses. Gingival fibroblasts were cultured and exposed to a tenfold dilution of selected oral rinses for 2 minutes. Afterwards, cells were stained with fourfold PBS diluted 0.4% trypan blue for 1 minute. The blue color specifies the disrupted cell membranes with increased permeability to the staining solution, indicating cell death. Oral rinses #1 to #5 show intact cell membranes, suggesting vital cells after exposure. Cells detached from the surface after exposure to oral rinse #6, and therefore fewer blue-stained cells were visible. All images were taken at a tenfold magnification.

To investigate the toxicity of oral rinses when applied to gingival fibroblasts, HSC-2, and L929 cells, a formazan formation assay was performed. The LC50 values were calculated—except for oral rinses #1 and #2, because formazan formation was unchanged (Figure 1). Also, the morphological and viability changes of gingival fibroblasts were negligible (Figure 2). In addition the cell membranes were intact indicating no toxic impact on the cells (Figure 3). Oral rinse #3 exhibited LC50 values > 50% with no visible changes in cell morphology. Oral rinses #4 and #5 exhibited LC50 values > 20% and were considered moderately toxic. The other seven oral rinses exhibited LC50 values < 20%, indicating severe cell toxicity (Figure 1). Exposed cells were round-shaped and detached (Figure 2). Thus, oral rinses can exhibit a broad spectrum of toxicity that ranges from negligible to severe in vitro cell cytotoxicity.

### 3.2. Antimicrobial Activity of Oral Rinses Containing Ethanol, CHX, CPC, PEG, SDS, SF, or Flavors

To investigate the antimicrobial effects of the oral rinses, screening tests were performed. The inhibitory zone diameter was examined following the application of each oral rinse to the following bacterial strains:* A. naeslundii*,* Strep. gordonii*,* P. gingivalis*,* F. nucleatum*,* T. forsythia,* and a mixed population of all five strains. We observed that oral rinses #1, #2, and #5 had no inhibitory effect on bacterial growth. Oral rinse #4 selectively inhibited bacterial growth as indicated by the inhibitory zone diameter of 12 mm observed for* T. forsythia* but not the other strains. Oral rinses #9, #11, and #12 produced inhibitory zones with a diameter < 15 mm, suggesting a moderate activity against bacteria. Oral rinses #3, #6, #7, #8, and #10 exhibited potent antimicrobial activity as observed in the inhibition zone diameters > 15 mm (Table 3). Overall, eight oral rinses showed a potent antimicrobial activity against oral bacteria.

### 3.3. Viability-Antimicrobial Plot of Oral Rinse Additives

**Figure 4:**
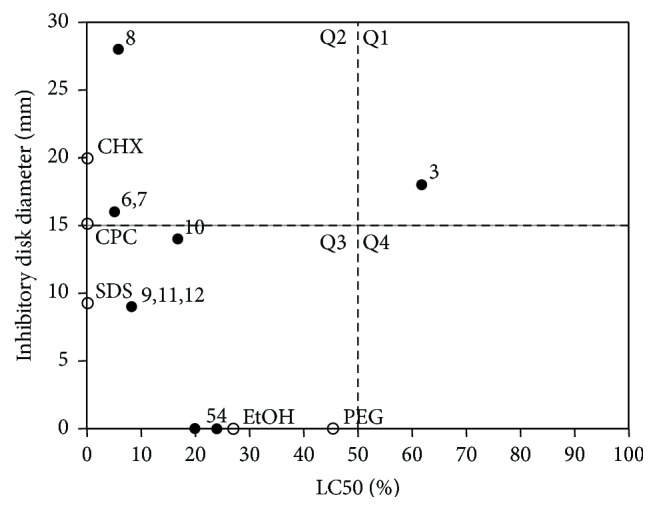
Lethal concentration (LC) 50 for oral fibroblasts and inhibitory zone diameter of a mix of microorganisms exposed to a series of oral rinse dilutions. Gingival fibroblasts cells were cultured overnight and exposed to selected oral rinse dilutions for 2 minutes (Table 1). Cell viability was measured with a formazan formation assay. Statistical analysis was performed, and LC50 was calculated. Additionally, a mix of bacteria, containing* Actinomyces naeslundii* ATCC 12104,* Streptococcus gordonii* ATCC 10558,* Porphyromonas gingivalis* ATCC 33277,* Fusobacterium nucleatum* ATCC 25586, and* Tannerella forsythia* ATCC 43037 strains, was cultured and exposed to the undiluted oral rinses, and the inhibitory zone was measured in millimeters after 40 hours. Oral rinse additives served as controls. Quadrant 1 (Q1) indicates good bacterial inhibition combined with low cell toxicity. Q2 shows high bacterial and cell viability inhibition. Q3 contains oral rinses with cytotoxic activity and low antibacterial effectiveness, and Q4 indicates low antibacterial and cytotoxic activity. For oral rinses #1 and #2 and SF (Tables 1 and 3), LC50 was not calculable because cells remained vital and no toxic effects were observed. Black circles indicate oral rinses and empty circles additives. For all experiments, cells with less than 10 passages were used. At least five independent experiments for each cell line were performed.

Additionally, the impact of oral rinse additives on cell viability and antimicrobial activity was assembled in a plot. Ethanol with a LC50 of 25.2% had no impact on bacterial growth (Figure 4). In addition, PEG (LC50 of 45.7%) and SF (LC50 not calculable) have no antimicrobial effect. CHX (LC50 0.02%) and CPC (LC50 0.06%) inhibited bacterial growth with high effectiveness, whereas SDS (LC50 0.36%) showed moderate antimicrobial effects in the selected concentration (Figure 4 and Tables 2 and 3).

### 3.4. Viability-Antimicrobial Plot of 12 Oral Rinses

Next, we prepared a plot to relate the viability data (LC50) with the antimicrobial activity (inhibitory zone diameter) of the oral rinses. Oral rinse #3 was located in Quadrant 1 (Q1) and represented low cytotoxic and high antimicrobial activities (Figure 4). In Q2, oral rinses #6, #7, and #8 represented both strong antimicrobial and strong cytotoxic activity (Figure 4). All other oral rinses were located in Q3, which indicates high cytotoxic and low antimicrobial activity. In contrast, oral rinses #1 and #2 exhibited no antimicrobial or cytotoxic activity (Figure 1 and Table 3). Thus, the spectrum of oral rinses tested was heterogeneous, with only some exhibiting both strong antimicrobial and strong cytotoxic activities.

## 4. Discussion

Here we investigated a dozen oral rinses with regard to their cytotoxic and antimicrobial effects on oral cells and bacteria. We discovered one oral rinse (#3) that exhibited low cytotoxicity but high antimicrobial activity. In contrast, other oral rinses were highly cytotoxic with no (#4, #5) or low (#9–#12) antimicrobial activity. All other oral rinses exhibited equal cytotoxic and antimicrobial activity, ranging from no (#1, #2) to high (#6–#8) activity. Thus, oral rinses are heterogeneous with regard to their antimicrobial and cytotoxic activities in vitro. So the question arises—are there component-related effects that cause this different antimicrobial and cytotoxic activities of the oral rinse? It might be based on the supplementation of active additives.

Oral rinses #9 and #10, which contained cetylpyridinium chloride (CPC), caused a similar response compared to CPC alone, namely, a highly cytotoxic and moderate antimicrobial activity. Thus, the findings with the respective oral rinses are presumably caused by CPC, the antimicrobial salt, and cationic surfactant [21]. Moreover, CPC is effective in preventing dental plaque and in reducing gingivitis [26]. CPC is described as being equally as effective as CHX in lowering the levels of bacteria [26]. Moreover, the FDA has considered oral rinses containing 0.1% CPC as safe for short-term use [22]. CPC, however, has exhibited potent in vitro cytotoxicity against human keratinocytes and L929 cells and potent antimicrobial properties [23]. Our data support the concept of CPC acting as a surfactant and an antimicrobial agent.

Oral rinse #8 was highly cytotoxic and exhibited potent antimicrobial activity. This observation reflected the findings observed with CHX alone and demonstrated strong cytotoxic and antimicrobial action. Oral rinse #3, containing CHX, however, showed rather low cytotoxic but robust antimicrobial activity. Thus, oral rinse #3 has a favorable ratio of preserving eukaryotic cell viability while inhibiting growth of prokaryotic bacteria. The reason might be the concentration of CHX, which is 0.2% and 0.05% in #8 and #3, respectively. CHX at low concentrations that do not substantially harm cells seems to be a potential antimicrobial compound [27–29]. The 15% ethanol of oral rinse #3 cannot be held responsible for the antimicrobial activity, as, in the present study, 20% ethanol failed to suppress bacterial growth. Our data point out that CHX is highly efficient in blocking bacteria growth even at concentrations that do not markedly impair cell viability.

Oral rinses #5, #7, #11, and #12 contain PEG, chemical softener, CAPB, or propylene glycol in combination with* Melaleuca alternifolia*, a tea-tree oil. PEG alone is nontoxic to cells and bacteria [30]. Interestingly, oral rinse #5, which contains PEG and propylene glycol, has a high cytotoxicity but no antimicrobial activity. Our data with PEG alone, being neither cytotoxic nor antimicrobiotic, cannot explain the effects of the respective oral rinses—except for the lack of antimicrobial activity of oral rinse #5. The in vitro cytotoxicity of #5 can be attributed to Melaleuca alternifolia and propylene glycol, an oil and a chemical solvent, respectively, that show cell death at high concentrations [31]. Oral rinses #7 and #12 contain softener, which is known for its toxic impact on cells and bacteria [32]. CAPB is a component of oral rinse #12 with the characteristic of a surfactant harming proeukaryotes and eukaryotes [33]. Thus, the high cytotoxicity of oral rinses #5, #7, #11, and #12 cannot ascribe to PEG, which is in accordance with our data with PEG alone. Therefore, the effects of #7, #11, and #12 can be credited to other ingredients, such as chemical softener and CAPB.

Other oral rinses containing SDS are #2 and #4, which also cause discrepancies. While oral rinse #2 failed to have cytotoxicity, oral rinse #4 is cytotoxic; both have a negligible antimicrobial effect. Considering our data that SDS alone is highly cytotoxic with a moderate antimicrobial activity, the findings obtained with the two oral rinses seem to be controversial. It might be that SDS in oral rinse #2 is so low that it does not even cause cell damage in vitro. Oral rinse #4 might have an SDS concentration that is sufficient to be cytotoxic but at least below 1%, which causes the moderate antimicrobial activity. Considering that oral rinse #4 also contains 21% ethanol—and 20% ethanol alone has moderate cytotoxicity but no antimicrobial activity—the effects of oral rinse #4 can be credited to ethanol.

The strong cytotoxic and antimicrobial activity of oral rinse #6 might be attributed to cocamidopropyl betaine (CAPB), an organic surfactant used in cosmetic and cleanser products, that provokes sensitization reactions on skin exposure [34]. CAPB has been attributed as the cause of irritation and dermal cell death [33, 35]. Moreover, CAPB suppresses the growth of proeukaryotes and eukaryotes via cell lysis, including Gram-negative bacteria and human cells [34, 36]. High doses of CAPB cause acute oral toxicity [33]. Together, the data support the concept that the cytotoxic and antimicrobial activity of oral rinse #6 is at least partially caused by CAPB.

Oral rinse #2 is supplemented with sodium fluoride, an inorganic chemical compound used to maintain dental health by the formation of fluorapatite, an integral component of tooth enamel after fluoridation [37]. Sodium fluoride is nontoxic to various oral cells and does not impact oral microbiota viability [38–40]. This is in line with our experiments that sodium fluoride was noncytotoxic and that bacterial growth remained unaffected. As long as oral rinses are not extensively swallowed, the usage of oral rinses supplemented with fluoride is safe.

The investigation on cytotoxicity is based on the monolayer cultures, which is a limitation of the study. The oral cavity contains cells from different origins and functions, including oral fibroblasts, epithelial cells, and immune system cells, which are stacked in multiple layers. An elegant model with epithelial cells to imitate the situation in the oral cavity was reported [41, 42]. Extrapolating in vitro results to the oral cavity remains difficult. Thus, the clinical relevance of the presented data is mainly attributed to the divergent cytotoxic and antimicrobial activities. While oral rinses used for cosmetic purposes do not necessarily have to be antimicrobial, the control of supragingival plaque and gingivitis as well as the need for oral decontamination before oral and periodontal surgery requires antimicrobial activity [2]. Oral rinses #1, #2, #4, and #5 should be considered cosmetic oral rinses as they lack antimicrobial activity. With regard to oral rinses with antimicrobial activity, those containing CHX (#3, #8) were more potent compared to those containing CPC (#9, #10) and those containing other components (#6, #7, #11, and #12). In general, oral rinses with antimicrobial activity are also cytotoxic. Based on the findings with oral rinse #3, it might be a viable approach to formulate a solution with high antimicrobial but low cytotoxic activity.

In summary the in vitro results of this study are heterogeneous in regard to the antibacterial and cytotoxic effect of oral rinses on oral cells. It is difficult to draw a clear dose-response based on the composition of oral rinses because the exact mixture is not public. In addition an international standard is not described to test and validate oral rinses based on their antibacterial and cytotoxic impact on the oral cavity. Here we elaborate a primary proof of concept study investigating the properties of oral rinses. Further independent research is necessary to postulate new standards of oral rinse formulation in regard to their medical purpose.

## 5. Conclusion

Oral rinses are heterogeneous with respect to their in vitro antimicrobial activity against bacteria and their effects on oral cell viability. We can recommend some preliminary suggestions on which components of oral rinses mediate the antimicrobial and cytotoxic activities, but since the exact composition of the commercial oral rinses remains unknown, the conclusions should be considered with caution.

## Figures and Tables

**Table 1:** Composition of the investigated oral rinses and oral rinse additives.

Product name	Manufacturer	CHX [%]	EtOH [Vol%]	SDS	PEG	CPC	CAPB	SF
#1 Mundspülung Hauschka	WALA Heilmittel, Germany		9					
#2 Emofluor	Dr. Wild, Switzerland			+				250 ppm
#3 Parodentosan oral rinse	Tentan, Switzerland	0.05	15					
#4 Listerine Total Care	Johnson & Johnson, UK		21	+	+			
#5 Tebodont Mundspülung	Dr. Wild, Switzerland				+			
#6 Dontodent	dm-drogerie markt, Austria						+	
#7 Meridol	GABA, Switzerland				+			250 ppm
#8 Meridol med	GABA, Switzerland	0.2						
#9 Sensodyne	GlaxoSmithKline, UK				+	+		217 ppm
#10 One drop only	One Drop Only, Germany				+	+		
#11 Elmex sensitive professional	GABA, Switzerland				+			250 ppm
#12 Elmex professional erosion	GABA, Switzerland				+		+	500 ppm

**(a):** 

	#1	#2	#3	#4	#5	#6	#7	#8	#9	#10	#11	#12
LC50	—	—	62.3%	23.7%	19.6%	7.0%	5.8%	6.9%	12.4%	11.9%	6.8%	7.4%

Gingival fibroblasts were exposed to a series of dilutions of oral rinses, and LC50 was analyzed after 2 minutes. Values show the percentage of dilution based on the original formulation of oral rinse. For oral rinses #1 and #2, more than 50% of the cells remained vital after exposure, and, therefore, no LC50 was analyzed, indicated with —.

**(b):** 

	EtOH	CHX	CPC	PEG	SDS	SF 500 ppm
LC50	25.2%	0.02%	0.06 mM	45.7%	0.36%	0%

Cell cultures of gingival fibroblasts were exposed to various oral rinse additives. LC50 was analyzed after 2 minutes of exposure time.

**(a):** 

	#1	#2	#3	#4	#5	#6	#7	#8	#9	#10	#11	#12
*Strep. gordonii*	0	0	17	0	0	14	13	24	7	13	9	9
*A. naeslundii*	0	0	19	0	0	16	15	28	7	15	9	7
*P. gingivalis*	0	0	38	0	0	25	19	28	11	21	11	10
*F. nucleatum*	0	0	28	0	0	33	—	33	7	—	7	7
*T. forsythia*	0	0	11	12	0	—	23	50	12	24	12	8
Mix	0	0	18	0	0	16	15	28	9	14	9	9

Bacteria were cultured and exposed to the undiluted oral rinses. The inhibitory zone diameter of the different bacterial cultures was measured in millimeters after 18–40 hours, indicating antibacterial properties (— indicates no growth).

**(b):** 

	EtOH 20%	CHX 0.2%	CPC 0.025 mM	PEG 10%	SDS 1%	SF 500 ppm
*Strep. gordonii*	0	15	13	0	8	0
*A. naeslundii*	0	19	11	0	9	0
*P. gingivalis*	0	23	14	0	8	0
*F. nucleatum*	0	23	11	0	8	0
*T. forsythia*	0	22	12	0	0	0
Mix	0	19	13	0	9	0

Bacteria were cultured and exposed to various oral rinse additives. The inhibitory zone diameter of the different bacterial cultures was measured in millimeters after 18–40 hours, indicating antibacterial properties.

## Abbreviations

CAPB: Cocamidopropyl betaine
CHX: Chlorhexidine
CPC: Cetylpyridinium chloride
EtOH: Ethanol
PEG: Pegylated hydrogenated castor oils
SDS: Sodium dodecyl sulfate
SF: Sodium fluoride.
